# Dexterous Control of Seven Functional Hand Movements Using Cortically-Controlled Transcutaneous Muscle Stimulation in a Person With Tetraplegia

**DOI:** 10.3389/fnins.2018.00208

**Published:** 2018-04-04

**Authors:** Samuel C. Colachis, Marcie A. Bockbrader, Mingming Zhang, David A. Friedenberg, Nicholas V. Annetta, Michael A. Schwemmer, Nicholas D. Skomrock, Walter J. Mysiw, Ali R. Rezai, Herbert S. Bresler, Gaurav Sharma

**Affiliations:** ^1^Medical Devices and Neuromodulation Group, Battelle Memorial Institute, Columbus, OH, United States; ^2^Neurological Institute, The Ohio State University, Columbus, OH, United States; ^3^Department of Biomedical Engineering, The Ohio State University, Columbus, OH, United States; ^4^Department of Physical Medicine and Rehabilitation, The Ohio State University, Columbus, OH, United States; ^5^Advanced Analytics Group, Battelle Memorial Institute, Columbus, OH, United States

**Keywords:** brain-computer interface, functional electrical stimulation, spinal cord injury, neuro-orthotics, functional hand grasping

## Abstract

Individuals with tetraplegia identify restoration of hand function as a critical, unmet need to regain their independence and improve quality of life. Brain-Computer Interface (BCI)-controlled Functional Electrical Stimulation (FES) technology addresses this need by reconnecting the brain with paralyzed limbs to restore function. In this study, we quantified performance of an intuitive, cortically-controlled, transcutaneous FES system on standardized object manipulation tasks from the Grasp and Release Test (GRT). We found that a tetraplegic individual could use the system to control up to seven functional hand movements, each with >95% individual accuracy. He was able to select one movement from the possible seven movements available to him and use it to appropriately manipulate all GRT objects in real-time using naturalistic grasps. With the use of the system, the participant not only improved his GRT performance over his baseline, demonstrating an increase in number of transfers for all objects except the *Block*, but also significantly improved transfer times for the heaviest objects (videocassette (*VHS*), *Can*). Analysis of underlying motor cortex neural representations associated with the hand grasp states revealed an overlap or non-separability in neural activation patterns for similarly shaped objects that affected BCI-FES performance. These results suggest that motor cortex neural representations for functional grips are likely more related to hand shape and force required to hold objects, rather than to the objects themselves. These results, demonstrating multiple, naturalistic functional hand movements with the BCI-FES, constitute a further step toward translating BCI-FES technologies from research devices to clinical neuroprosthetics.

## Introduction

Approximately 130,000 people suffer a Spinal Cord Injury (SCI) worldwide every year. Nearly half of these SCI cases are at the C6 level or above, resulting in significant paralysis, impaired quality of life, and need for self-care assistance (ICCP, 2017). Moreover, patients with C6 or higher cervical level of SCI lack the critical ability to grasp objects that prevents them from living independently (Nas et al., 2015). Indeed, several studies on SCI patient priorities have consistently reported that upper limb strength and dexterity restoration is the most desirable function to regain (Anderson, 2004; Snoek et al., 2004; Simpson et al., 2012; Collinger et al., 2013; Blabe et al., 2015). In a survey of individuals with tetraplegia following SCI, more than 75% indicated that Functional Electrical Stimulation (FES) neuroprosthetics for hand grasp would be “very helpful” to restore function that would positively impact quality of life (Collinger et al., 2013). However, the FES systems that have been demonstrated to date are either limited to providing only a few hand functions or lack the ability to enable dynamic motor control for performing complex functional tasks that require synergistic integration of paralyzed and non-paralyzed muscles.

Advances in Brain Computer Interface (BCI)-controlled FES technology offers a potential new way to reconnect the brain directly to the paralyzed hand/arm, restoring functional hand use. FES devices with control mechanisms other than BCI (e.g., myoelectric, sip-and-puff, eye trackers) have been proposed, but are less desirable due to increased cognitive load and non-intuitive mapping between thought and action (Ajiboye et al., 2017). Thus, BCI approaches are preferred for their ability to provide a more intuitive and “high-fidelity” control signal that can allow for more complex and clinically-relevant functional limb movements (Chadwick et al., 2011; Ethier and Miller, 2015). Indeed, in recent surveys a majority of paralyzed patients showed interest in using a BCI technology that can help restore lost hand/arm function (Collinger et al., 2013; Blabe et al., 2015).

Several groups have investigated BCI-FES neuroprosthetics for restoring hand grasp function in paralyzed humans with varied success. Some groups have coupled an electroencephalogram (EEG)-BCI with FES systems and showed that the paralyzed participants were able to use the systems to enable up to two functional hand movements by imagining hand/arm movement (Müller-Putz et al., 2005) or by imagining a non-intuitive motion such as foot (Pfurtscheller et al., 2003) or cursor movement (Lauer et al., 1999). However, the low dimensional control signals of the EEG as well as non-intuitive mapping of thoughts-to-action makes it unlikely that these BCIs could provide naturalistic continuous control for complex hand functions. An alternative approach, utilizing electrocorticography (ECoG)-based signals, can provide better spatial resolution compared to EEG and thus a potential neuroprosthetic control mechanism based on high quality neural signals. Indeed, a paralyzed participant using an ECoG-BCI controlled transcutaneous FES system was successfully able to perform three movements (hand open, palmer, and lateral grasps) (Márquez-Chin et al., 2009). However, this demonstration was done in an offline mode where ECoG signals recorded from an able-bodied participant were used to control FES-evoked movements of the paralyzed participant. Therefore, the applicability of ECoG-BCI for real-time control of multiple hand movements via FES orthotics remains to be demonstrated. To overcome the limitations of EEG/ECoG control, researchers have implanted intracortical microelectrode arrays (MEAs) that can allow for higher information transfer rate (Baranauskas, 2014) and a more precise detection of movements for decoding and controlling hand/arm FES systems. In a prior study, we showed proof-of-concept that a person with C5-level paralysis could use a MEA-BCI to control a transcutaneous FES system to enable six independent finger, wrist, and hand movements (Bouton et al., 2016) We also demonstrated that the system could be used to perform a functional grasp-pour-and-stir task, providing the user with simultaneous, differential control of Hand Open, palmar grasp, and lateral key grip. A similar study showed proof-of-concept that a person with C4-level paralysis could use a MEA-BCI to control a hybrid exoskeleton and implanted FES system to evoke upper limb reaching, Hand Open, and lateral key grip (Ajiboye et al., 2017). The participant in this study used these movements to perform functional feeding tasks. However, no prior study has provided careful quantification and characterization of MEA-BCI enabled FES upper limb motor control to allow for study reproducibility and comparison with other neuroprosthetic devices.

**Figure 1:**
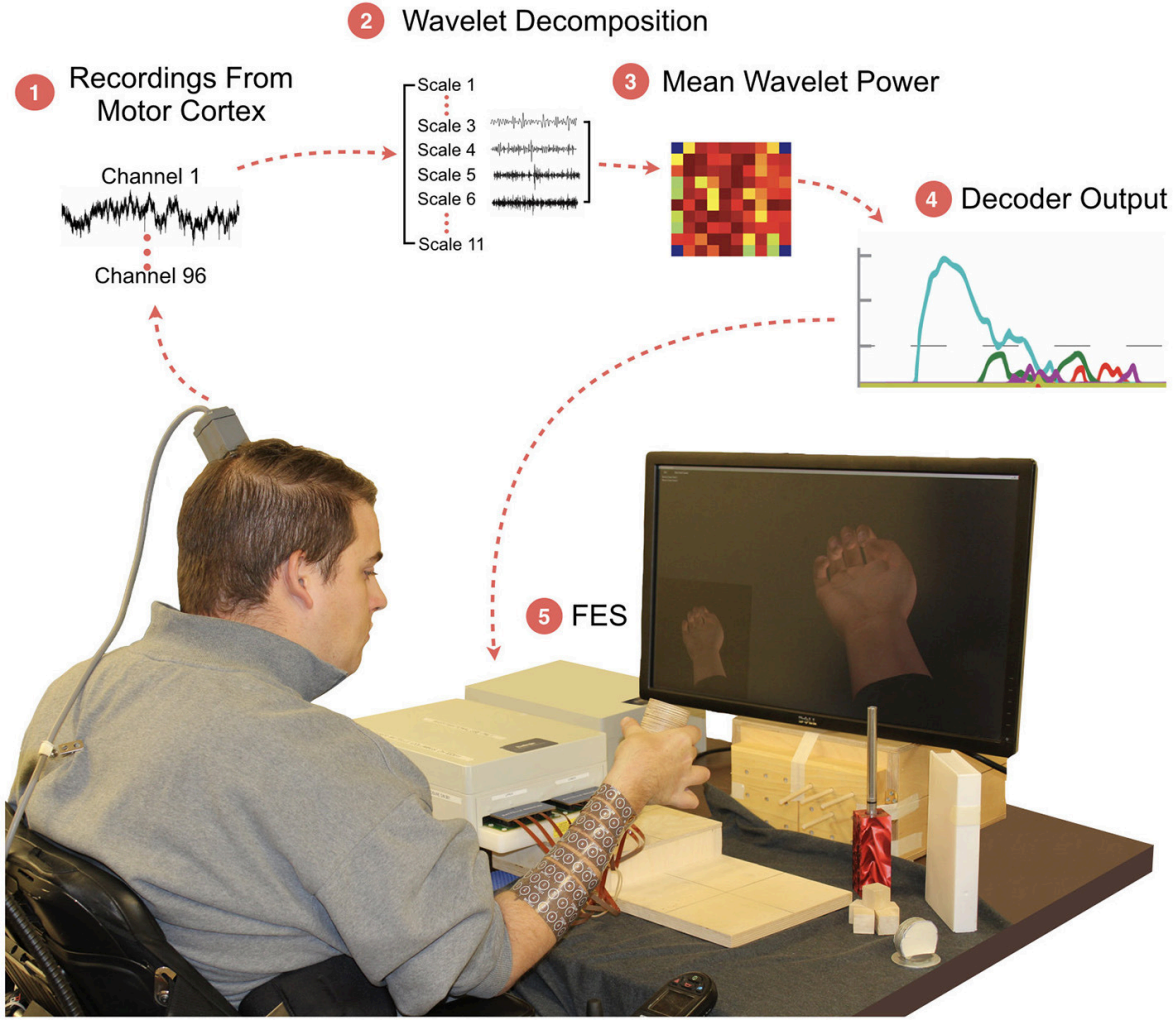
The BCI-FES system and experimental setup. The participant sits on wheelchair in front of the monitor which shows him the cued hand movement. The participant is required to grasp and transfer the object to the raised platform. (1) Neural activity is recorded from a 96-channel MEA implanted in the motor cortex; (2) A wavelet decomposition is performed on the raw data to extract neural information related to motor intent; (3) Wavelet scales 3 through 6 are used to generate Mean Wavelet Power (MWP)-based neural features; (4) Machine-learning algorithms decode the MWP activity for each attempted hand movement; (5) Hand movement is evoked using targeted transcutaneous FES delivered through cuffs wrapped around the forearm.

In this study, we show a critical step in the clinic-to-home translational path of BCI-FES neuroprosthetics by demonstrating that a patient with tetraplegia can achieve volitional control of seven hand functions using an easy to train, cortically-controlled, non-invasive, FES orthotic. We used a MEA, implanted in the motor cortex of a 26-year old study participant with a C5-level SCI, to record neural signals. We then used machine learning algorithms to translate the neural activity to intended movement commands. These commands were then used to control the transcutaneous FES orthotic wrapped on the participant's forearm which stimulated the appropriate muscles to evoke the intended movement (Figure 1). With the system, the participant was able to use a trained decoder to volitionally select up to seven distinct functional hand states and use them to manipulate multiple objects of varying size, shape, and weight. The participant's functional gains were assessed using the Grasp and Release Test (GRT; Stroh-Wuolle et al., 1994), a standardized test developed for evaluating neuroprosthetic performance by patients with SCI. We found more efficient grasp and transfer of objects using the BCI-FES compared to the participant's baseline. Our results also revealed important insights into the neural representation of different hand movements. In particular, we observed that a robust mapping of multiple hand movements can form under the implanted MEA in a very small area of the motor cortex. We found overlap between representations for objects of similar size and weight and we report a strong correlation between the discriminability in the neural representations of hand movements and decoder performance.

## Materials and methods

### Study design and study participant

The objective of this study was to characterize the level of upper limb motor control provided by a cortically controlled FES system in a patient with SCI. A secondary aim was to investigate the neural representations underlying grasps used for different objects. The study was approved by the US Food and Drug Administration (FDA) and The Ohio State University Wexner Medical Center Institution Review Board (Columbus, Ohio) and is registered on the ClinicalTrials.gov website (Identifier NCT01997125). The participant referenced in this work provided permission for photographs and videos and completed a written informed consent process prior to commencement of the study. The participant is a 26-year-old male with stable, non-spastic tetraparesis from a cervical SCI that he suffered at the age of 19. His use of the BCI-FES system was first reported in Bouton et al. (2016). The participant's International Standards for Neurological Classification of SCI neurologic level is C5 AIS A (motor complete) with zone of partial preservation to C6. He has full active range of motion in bilateral shoulders, full bilateral elbow flexion, a twitch of wrist extension (insufficient for tenodesis grip), and no motor function below the level of C6. His sensory level is C5 on the right (due to altered but present light touch on his thumb) and C6 on the left. He has intact proprioception in the right upper limb at the shoulder for internal rotation through external rotation, at the elbow for flexion through extension, at the forearm for pronation through supination, and at the wrist for flexion through extension. Proprioception for right digit flexion through extension at the metacarpal-phalangeal joints is impaired for all digits.

### System architecture

**Figure 2:**
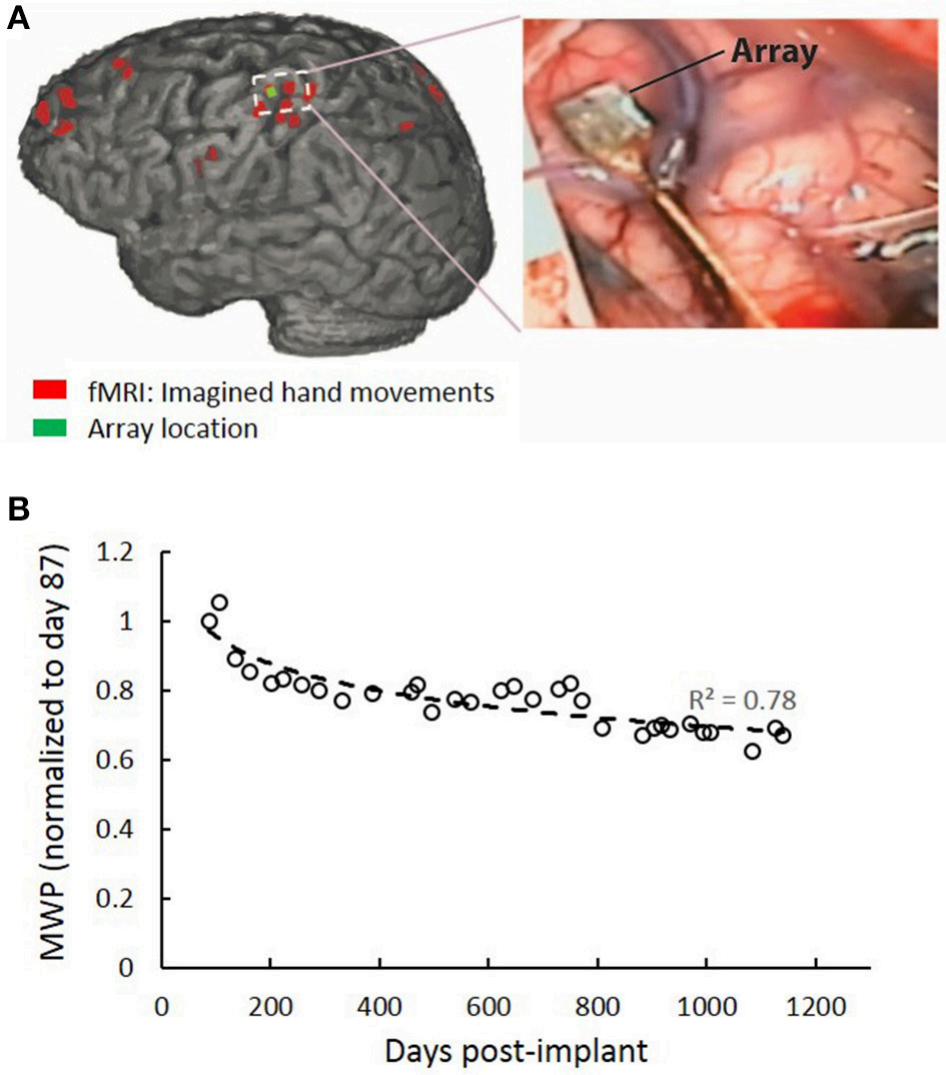
MEA location and signal quality over time. **(A)** Red regions are brain areas active during imagined hand movements. The implanted MEA location from post-op CT is shown in green. **(B)** MWP data for all channels were collected over a 108 s period at the beginning of periodic test sessions where the participant was instructed to imagine cued hand movements. MWP features were calculated to approximate the power in the multiunit frequency bands a plotted as a function of post-implant days. A 33% decline in the signal quality was observed over time from the MWP data.

The system is comprised of three main components: (i) A Utah Microelectrode Array (MEA) implanted in the hand region (identified using preoperative fMRI activation maps) of the left-brain hemisphere motor cortex and a Neuroport neural data acquisition system (Blackrock Microsystem Inc., USA). Figure 2A shows the implant location in the motor cortex which was confirmed by co-registration of postoperative computed tomography (CT) imaging with preoperative fMRI. Full details of the fMRI and surgical procedures can be found in Bouton et al. (2016), (ii) a computer running data processing and machine learning algorithm to decode the user's intended movement from the neural activity, and (iii) A custom high-definition non-invasive FES system with 130 electrodes used to stimulate the hand/arm muscles to evoke desired hand movements. The stimulator was driven by custom MATLAB (ver 2014b, MathWorks Inc., USA) based code running on a PC.

### Neural data acquisition and signal processing

The 96 MEA channels recorded the electrical activity in the cortex at a sampling rate of 30 kHz. The raw voltages were first filtered using a 0.3 Hz first-order high-pass filter and a 7.5 kHz third-order low-pass Butterworth analog hardware filter. Wavelet decomposition using the “db4” wavelet and 11 wavelets scales was applied to the neural data in 100 ms bins (Mallat, 1998). Wavelet scales 3–6 were used, corresponding to the multiunit activity (MUA) (234–3,750 Hz). The mean coefficients of scales 3–6 were standardized per channel, per scale, by subtracting the mean and dividing by the standard deviation of those scales and channels, respectively. The four scales were then combined by averaging the standardized coefficients for each channel, resulting in 96 values, one for each channel of the MEA, for every 100 ms of data. The resulting values were subsequently used as features, termed mean wavelet power (MWP), for input into the real-time decoders. Stimulation artifact in the data was removed by first applying a threshold at 500 μV that occurred simultaneously on at least 4 of 12 randomly selected channels. A 3.5 ms window of data encompassing each detected stimulation artifact was then removed and adjacent data segments were concatenated. To look at MWP signal quality over the study period, data for all channels were collected over a 108 s period at the beginning of periodic test sessions where the participant was instructed to imagine cued hand movements. MWP features were calculated to approximate the power in the multiunit frequency bands. We observed a 33% decline in the signal quality over time (Figure 2B).

Threshold crossings (TCs) were calculated by filtering the raw voltage recordings through a 250 Hz high pass filter, using the filtered data to determine the root-mean-square (RMS) value of the noise (defined by Blackrock Microsystems, Inc.), and then applying a threshold of −4.5 times the RMS of the noise to the voltage recording. The data was not spike sorted. Approximately 86 and 27 TC spikes could be detected on post-implant days 87 and 1,144, respectively, during the same 108 s test period as described above. Correlation between average MWP and TCs was calculated during the first 55 s of a representative training block. Average MWP was calculated by averaging MWP across channels. Global TCs were calculated by binning TCs for all channels in 100 ms bins.

### Neural decoding

A non-linear Support Vector Machine (SVM) decoder (Humber et al., 2010) was used to translate the MWP activity to intended hand movements. The decoder was trained in blocks consisting of multiple repetitions of all desired movements. Output classes were built for each movement and had scores that ranged from −1 to 1. Appropriate stimulation became activated when an output score of a given movement exceeded a threshold of zero. If multiple movement decoder output scores surpassed the threshold, the system enabled stimulation for the movement with the highest score. Individual movement accuracy was calculated from final training blocks as the percentage of 100 ms time points in which the decoder output for the given movement correctly matched the associated cue. Response probability for each cue (represented as a confusion matrix) was calculated from final training blocks as the percentage of activation for a single movement decoder class out of all active movement decoder classes within a cue. Individual movement accuracy scores and response probabilities were averaged across sessions of the same type. The final blocks of each training session were used for training the decoders. This was done to minimize the potential for muscle fatigue associated with repetitive FES of the same movements over a short period of time, which would have been required if we performed extra training blocks to measure decoder accuracy.

### Stimulation

The FES system consists of a multi-channel stimulator and a flexible cuff with up to 130 electrodes that is wrapped around the participant's forearm. During use, hydrogel disks (Axelgaard, Fallbrook, CA) were placed between the electrodes and skin to act as a conduction enhancer. The electrodes are 12 mm in diameter and were spaced at 22 mm intervals along the longitudinal axis of the forearm and 15 mm intervals in the transverse direction. Current-controlled, monophasic rectangular pulses (50 Hz pulse rate and 500 μs pulse width) were used to provide electrical stimulation. Pulse amplitudes ranged from 0 to 20 mA and were updated every 100 ms. Stimulator calibrations were performed for each movement using an anatomy-based trial-and-error method to determine appropriate electrode spatial patterns.

### Experimental design

The study sessions with the participant were typically conducted two or three times per week, lasting 3–4 h. Data used for this study were collected from eight sessions as follows: baseline GRT data on post-implant days 702 and 703; BCI-FES data on post-implant days 855, 857, 869, and 897; and imagined GRT data on post-implant days 1,042 and 1,043. The participant had prior experience using the BCI-FES system for other studies as reported in Bouton et al. (2016), Sharma et al. (2016), and Friedenberg et al. (2017). Sessions began with stimulation pattern calibrations for each hand movement. Stimulation patterns and intensity levels were saved in a database. In subsequent sessions with the participant, the previous calibrations were recalled and refined, if necessary. Calibrated movements included: (i) Index finger and thumb lateral key pinch for gripping a *Peg*, (ii) middle finger, index finger, and thumb tripod grip for gripping a *Block*, (iii) middle finger and thumb lateral key grip for gripping a *Paperweight*, (iv) ring finger and middle finger cylindrical power grip for gripping a depressible *Fork*, (v) tip-to-tip grip for gripping a videocassette (*VHS*), (vi) palmar power grip for gripping a *Can* (customized wooden cylinder), and (vii) finger and thumb extension (*Hand Open*) to open the hand. All objects used in this study conformed to specifications of the Grasp and Release Test (Stroh-Wuolle et al., 1994).

#### Neural decoder training

Training data for the decoder was obtained by prompting the participant to imagine performing specific hand movements using an animated virtual hand displayed on a computer monitor. During the cue duration, FES feedback allowed the participant to grasp the cued object in the starting area and transfer it to an elevated platform using the system. In the case of the *Fork* grip, the participant gripped the cylindrical handle of the *Fork* and applied downward pressure to displace a calibrated spring. Additionally, during cued *Hand Open*, the participant opened his hand by extending his digits. Each movement cue had a random duration between 3 and 4 s and was bounded by rest cues with random durations between 4 and 5 s. The ordering of the movement cues was randomly shuffled to eliminate cue anticipation. Each training block included 3 cues for each movement.

#### Grasp and release test (GRT) with FES

Functional grasps were assessed using the GRT (Stroh-Wuolle et al., 1994). The participant was presented with random, auditory cues for the different objects and was required to grasp the object in the starting area, lift and transfer the object to an elevated platform, and release the object in the target region as many times as possible in a 30 s test period. The participant was given a rest period of around 30 s between each 30 s test period. Dropping the object (or insufficient cylinder displacement for the *Fork*) was counted as a failure. The number of successful transfers, failed transfers, and incomplete transfers along with the associated transfer times for each object were recorded. For the *Fork*, successful “transfers” were counted if the spring-loaded piston was sufficiently displaced, indicated by a line on the piston. Two decoder classes were required for the *Can* transfer. The participant had to perform a *Hand Open* to position his hand in an optimal location around the *Can* and then initiate the *Can* grasp. During each cue, all movement decoder classes (seven possible) had equal potential to cross threshold and evoke FES stimulation. The GRT was performed 3 times per session for each object, with mean successful, failed and incomplete transfers reported per object and session. GRT testing was conducted over 4 sessions (for a total of 12 trials) for *Peg, Block, Paperweight, Fork*, and *VHS. Can* data was collected over 3 sessions (for a total of 9 trials). Test sessions were performed on post-implantation days 855, 857, 869, and 897.

#### GRT without FES

To visualize the neural representation of hand movements in the motor cortex, MWP activity was examined during cued movements without any FES or movement feedback. Both movement and stimulation can create artifacts that can alter the MWP despite efforts to filter them. Thus, FES was turned off during the test blocks to remove the potential confounding effects of artifacts from the analysis. Three independent blocks of trials per object were conducted using decoders built as described in the Neural Decoder Training section in Methods, except that feedback was provided using only the animated hand and not FES. The subject was instructed to place his hand on the cued object and then imagine performing the grasp. This dataset was collected over 2 consecutive sessions (days 1,042, 1,043 post-implantation). The MWP spatial patterns were compared using a Principal Component Analysis (PCA) applied to the MWP on all 96 channels when the correct decoder outputs were above threshold and within the correct cue durations. Principal components 1 and 2 were used to determine clustering. Each cluster was fit with a Gaussian mixture distribution model for visualization purposes. For each movement, MWP was averaged across all blocks when the associated decoder was above threshold and within the correct cue duration. The average MWP at each channel was spatially mapped to the physical layout of the MEA and displayed as a heat map. Finally, to quantify the separation between MWP spatial patterns, Euclidian distances between each movement's vectorized spatial pattern were calculated. The MATLAB Pairwise Distance (pdist) function was used for this analysis. Euclidian distances for each movement compared to all others were summed to determine the amount of separation in neural representation.

### Statistical analysis

Paired comparisons between total number of transfers and object transfer times for the GRT were performed using a paired *t*-test. Correlations between MWP similarity and decoder performance was assessed using a linear regression model. Correlations between TC and MWP were assessed using Pearson's correlation method. All statistical analyses were performed using MATLAB (ver 2014b) and *P* < 0.05 was considered statistically significant. Results are presented as Mean ± Standard Deviation (*SD*).

## Results

The cortically controlled FES system consisted of three main components: (1) an implanted 96-channel Utah MEA for recording neural signals, (2) a computer running data processing and a machine learning algorithm to decode the user's intended movement from the neural activity, and (3) a non-invasive FES cuff wrapped on the participant's forearm to stimulate the appropriate muscles to evoke the desired hand movement (Figure 1). Wavelet decomposition was used to process the raw cortical data into MWP neural features (see section Methods). These features were used as inputs to a SVM decoding algorithm that translated the neural activity to the user's intended movement, which was then used to control the electrical stimulation of the user's forearm (Figure 1). No device-related adverse events occurred during the duration of this study.

### Performing functional hand movements with high accuracy

**Figure 3:**
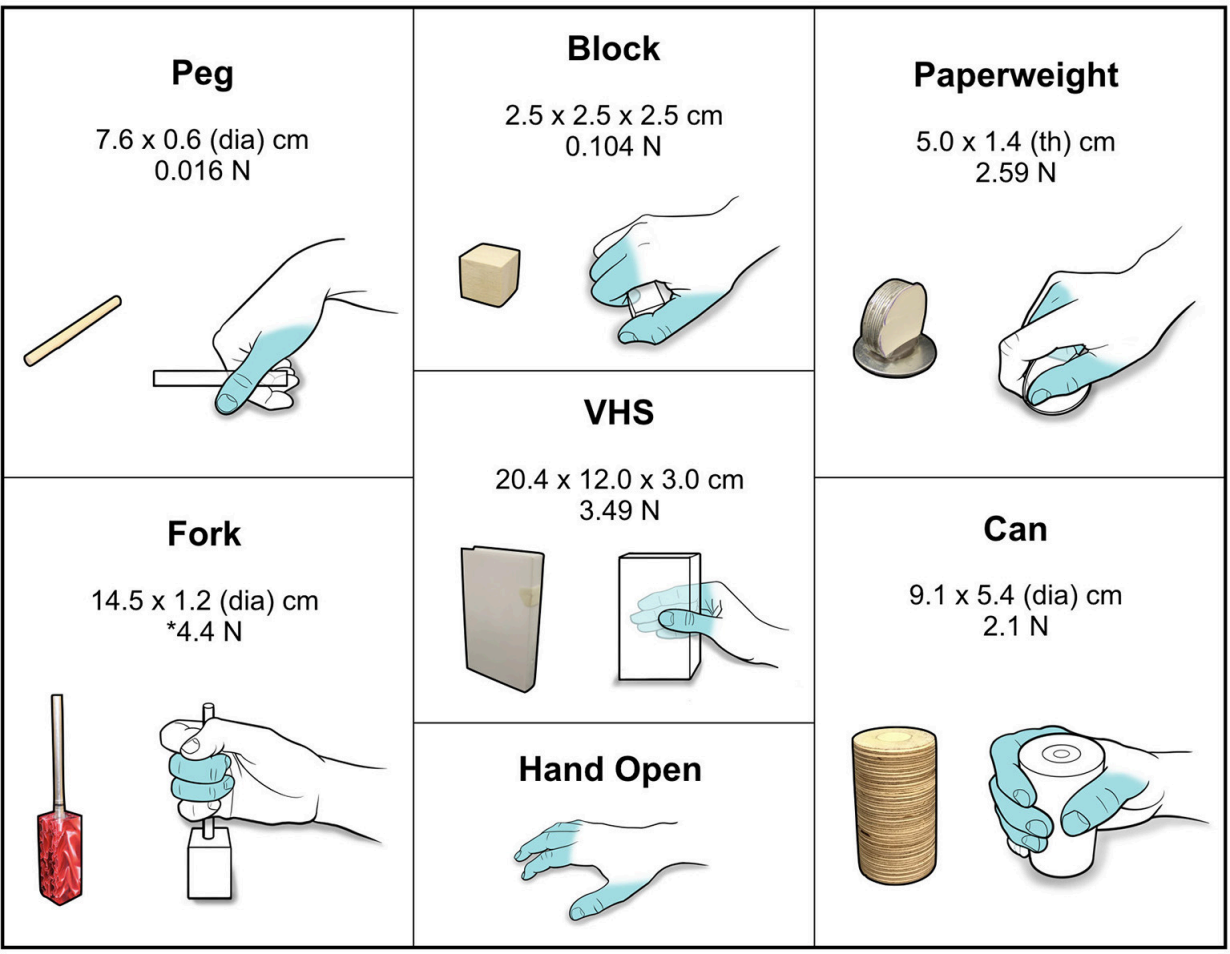
Standardized GRT objects and functional grasps. Schematic showing the different GRT objects with associated dimensions and weights. Hand schematics illustrate the grasp/movement enabled by FES for the object. Fingers that were activated and used to perform the grasps/movements are highlighted in blue. ^*^For the *Fork* object, a 4.4 N force is required to depress the cylinder.

Using the BCI-FES system, the subject was trained to perform seven distinct functional hand movements that were specific to grasp, transfer, and release of standardized test objects. The objects conformed to specifications for the GRT (Stroh-Wuolle et al., 1994) and are described in Figure 3. The FES system was calibrated to evoke seven discrete, dynamic hand states which included a specific grasp for each of the six GRT objects and a *Hand Open* movement (see Figure 3 for grasp schematics and Figure S1 for stimulation parameters and targeted forearm muscles groups for enabling each hand movement).

**Figure 4:**
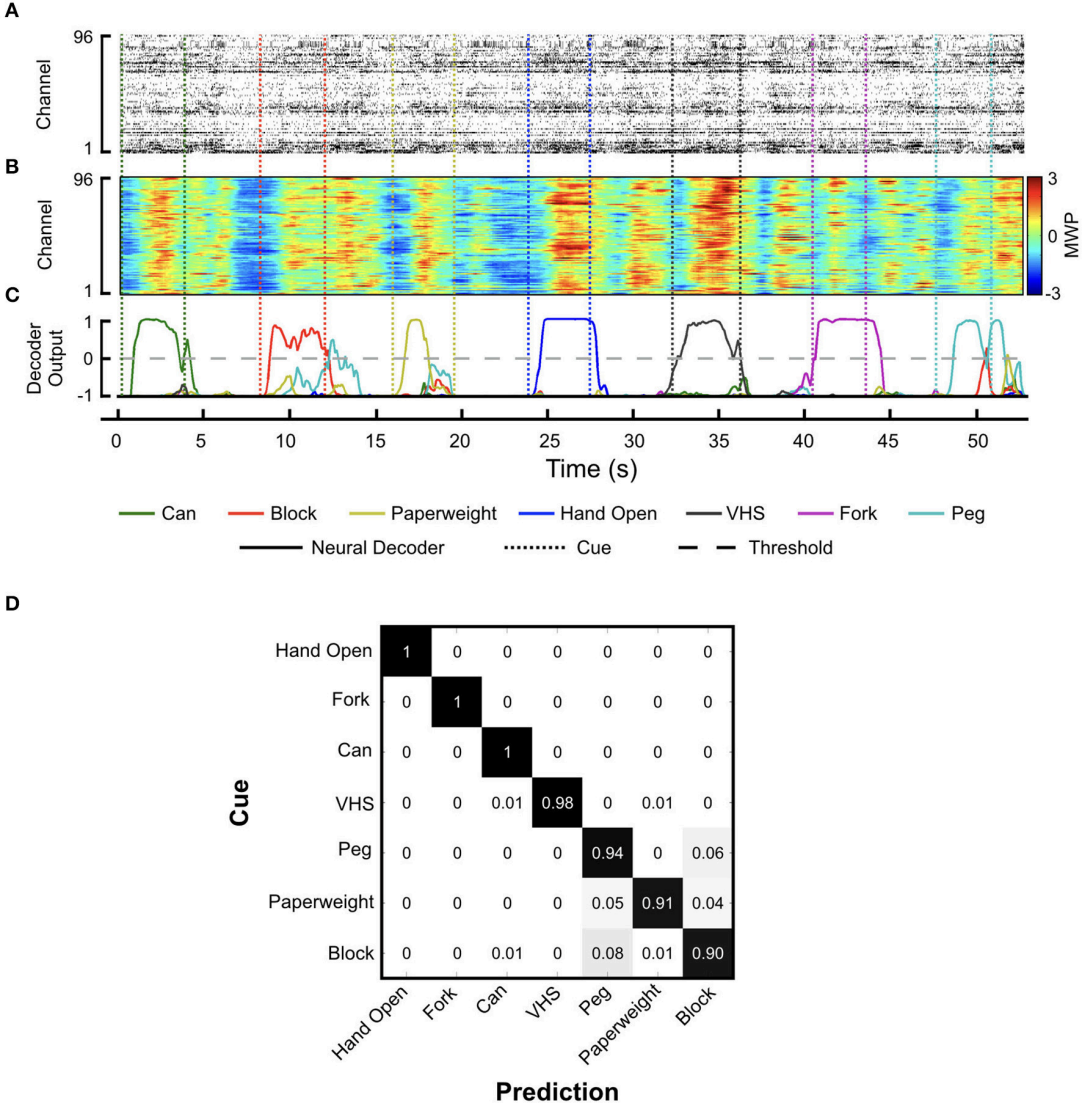
Neural decoder training. Representative plots showing **(A)** threshold crossing raster plot, **(B)** the corresponding MWP activity across all channels of the MEA, and **(C)** neural decoder output as the participant attempts the seven cued hand movements. Solid lines indicate neural decoder output and dotted lines indicate the cue start and stop times. Of the seven possible hand movement states that can be predicted by the decoder, the output score from the one with the highest amplitude greater than zero was used to turn on/off the stimulation; **(D)** Confusion matrix showing the decoder response probability for each movement cue.

During training, the participant received visual cues to initiate and terminate each hand movement interleaved with rest periods. Figures 4A,B shows a snapshot of the neural activity showing the MWP modulation and the corresponding threshold crossing (TC) neural activity raster plot. We observed a strong correlation between TC and MWP neural activity (correlation coefficient = 0.65, *p* < 0.001). The full set of MWP data was used as input for training and generating the neural decoder. Figure 4C shows representative decoder outputs during training as the participant attempted hand movements to manipulate the objects. When the decoder output for a particular movement crossed the zero threshold, the system initiated the FES to evoke the corresponding hand movement. The decoder was trained in 3-min blocks and it took 4–5 blocks of training (12–15 min of total training time) to generate a robust decoder set that could successfully classify seven hand movements for grasp, transfer, and release of different objects. Movie S1 shows the participant manipulating the randomly cued objects during training. Figure 4D depicts the confusion matrix showing the probability of the decoder classifying each hand movement. The results indicate that, in general, the predicted hand movement was correctly classified as the cued hand movement. The grips for *Hand Open, Fork*, and *Can* were always predicted correctly with response probabilities of 1. However, the decoder had more difficulty discriminating between the *Peg, Paperweight*, and *Block* grips (response probabilities = 0.94, 0.91, and 0.90, respectively). Overall, across all trials, the individual accuracy for decoding each movement ranged from 96.3 ± 0.7% (*Paperweight*) to 99.0 ± 0.5% (*Hand Open*) demonstrating the system's ability to correctly classify the imagined movement from the eight possible hand states (seven hand movements and a rest) (Table 1).

**Table 1:** Individual decoder accuracy.

**Hand movement**	**Individual decoder accuracy (%)**
Hand open	99.0 ± 0.5
Can	96.9 ± 0.9
Block	97.4 ± 1.2
Peg	96.9 ± 0.6
Fork	98.4 ± 0.8
Paperweight	96.3 ± 0.7
VHS	98.1 ± 0.2

*Individual decoder accuracies were calculated by determining the percentage of time points that the decoder output for a given movement correctly matched the associated cue during decoder training. Values presented as mean ± S.D*.

### Quantifying the gains in functional performance using the BCI-FES system

**Figure 5:**
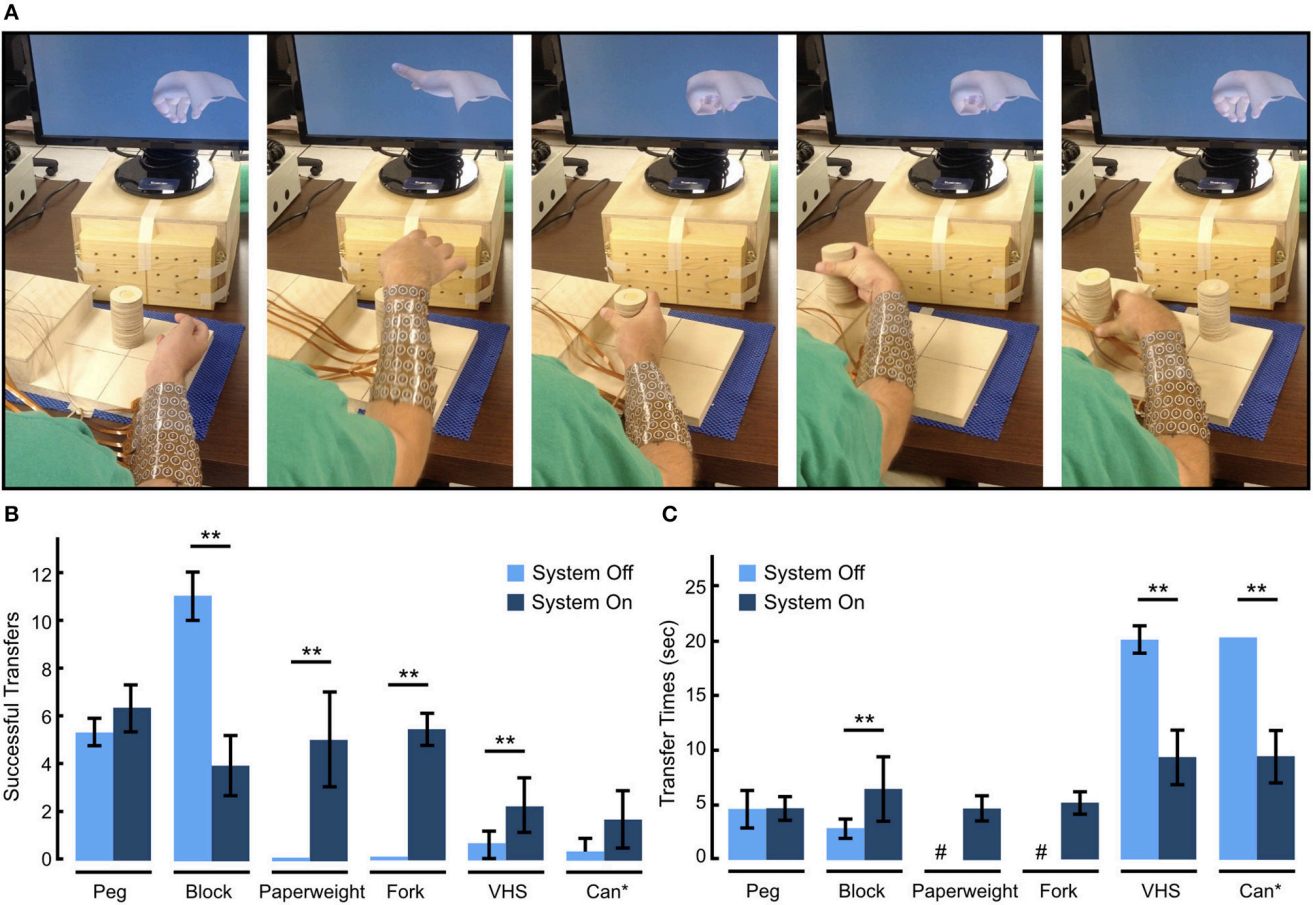
Functional performance evaluation using the Grasp and Release Test (GRT). **(A)** Sequential snapshots of the participant manipulating the *Can* object as part of the GRT. The participant starts from a rest state, opens his hand and place it around the *Can*, grasps the *Can*, transfers it to the raised platform, and then releases the *Can*. A new object is then placed in front of the participant to attempt the next transfer. **(B)** GRT scores showing the mean number of successful transfers with and without the BCI-FES system. With the use of the system, the participant not only improved his GRT scores over his baseline, demonstrating an increase in number of transfers for all objects except the *Block*, but was also able to grip and transfer two objects (*Paperweight, Fork*) that he could not manipulate at baseline. **(C)** Mean transfer times for each object with and without the BCI-FES system. With the use of the system the participant's transfer speed increased for all objects except for the *Peg* and *Block* which he was able to transfer faster on his own using adaptive grips. ^#^The participant was never able to transfer these objects without the system. ^*^The *Can* transfer required two hand movements—*Hand Open* and *Can* grasp. ^**^p < 0.05 (paired *t*-test).

A board-certified physiatrist administered the GRT (see section Methods) to investigate the participant's ability to use the BCI-FES system to manipulate objects across a range of sizes, shapes, and weights. In addition to providing standardized test objects, the GRT also allowed us to compare the performance of our system with others' who have used this test to investigate their BCI-FES systems. Figure 5A shows representative snapshots of the participant transferring the *Can* object as part of the GRT. To complete one transfer, the participant used voluntary shoulder movements to align his hand above the *Can*, initiated a *Hand Open* movement to extend his fingers and position the *Can* in his palm, then initiated and maintained a palmar grasp while he transferred the *Can* laterally to a raised platform, and finally, terminated the grasp to release the object from his hand. Movie S2 shows the participant manipulating the objects during one GRT block.

Figure 5B summarizes the participant's ability to manipulate GRT objects with and without use of the BCI-FES system. At baseline (Day 702–703 post-implant) and without the BCI-FES system, the participant could not efficiently manipulate (average number of successful transfers < 1) the *Paperweight, Can, VHS*, and *Fork*. However, he was able to grasp and manipulate the *Block*, and *Peg* using adaptive grip strategies. With the BCI-FES system (days 855–897 post-implant), the participant was not only able to evoke the correct hand movement from the possible eight states available to him, but was also able to successfully grasp, transfer and release all objects and successfully depress the *Fork* multiple times in the 30 s test period. In general, with the use of the system, the number of successful (failed) transfers increased (decreased) over baseline (See Figure S2 showing total number of failed transfer attempts with and without the use of the system). The *Block* was an exception, where the participant had fewer successes with the BCI-FES system than without, as the participant was able to use an adaptive grasp to transfer the *Block* on his own. In addition to being able to rapidly transfer the *Paperweight* (transfer time = 4.7 ± 1.2 s) and displace the *Fork* (displacement time = 5.1 ± 1.1 s) with the BCI-FES system, which he was otherwise not able to do on his own without the system, the participant also showed significant improvement in transfer times with the system for the other two heavier objects, i.e. the *VHS*, and *Can* (Figure 5C). However, it took the participant significantly longer to complete the *Block* transfer with the BCI-FES system (6.4 ± 3.0 s per *Block*) than without (2.8 ± 1.0 s per *Block*), while there was no significant change in the completion time for the *Peg* transfer.

### Investigating the correlation between neural discriminability and decoder performance

**Figure 6:**
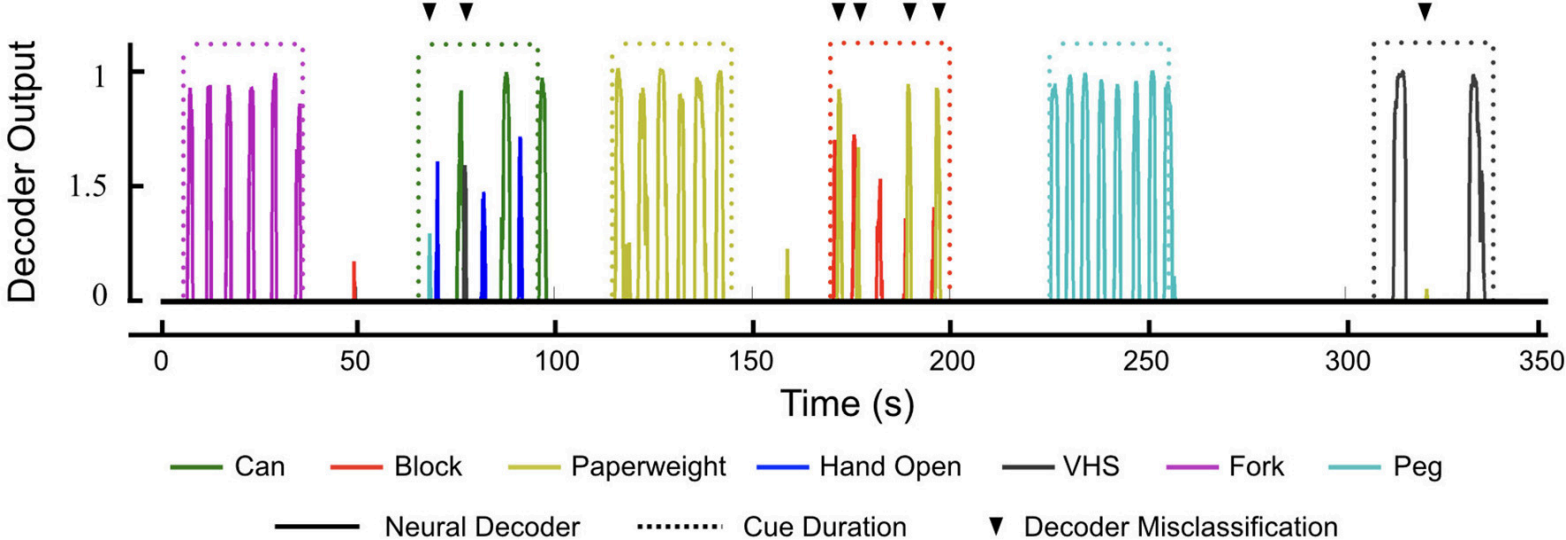
Neural decoder outputs during the GRT. Representative decoder outputs during a GRT test block showing instances of decoder misclassification (black triangles). All seven hand movements are available to the participant as part of the decoder and he has to evoke the correct movement (solid lines) during the 30 s trial period (dotted lines) given to him to complete the GRT for that object. Only decoder outputs above the activation threshold of zero are shown for visual clarity. Successful transfer of *Can* required the participant to evoke two hand movements—*Hand Open* and *Can* grasp (70–100 s). During the *Can* transfer, the decoder had two misclassifications (one of each for the PEG and VHS grasps). However, the participant was able to evoke the correct hand movements to successfully complete two *Can* transfers during the trial period. Similarly, during the *Block* transfer (170–200 s), the participant incorrectly evoked the decoder for *Paperweight* on four occasions. This did not affect the GRT scores for the *Block*, however, as the decoder for *Paperweight* kicked in after the participant had completed the *Block* transfer.

As the participant performed the GRT, we observed a few instances of decoder misclassification. In particular, the decoder would sometimes trigger the *Paperweight* grip when the participant tried to release the *Block*. Similarly, the *VHS* grip was sometimes evoked during the *Can* release. Figure 6 shows a representative decoder output plot from an entire GRT test block that provides examples of decoder misclassification. The participant was cued to transfer the *Block* beginning at 170 s. While the *Block* grip was correctly triggered for each of the transfers, the *Paperweight* grip was also incorrectly evoked 4 out of 5 times after the *Block* grip (see Movie S2 which shows the *Block* transfers during this test period).

**Figure 7:**
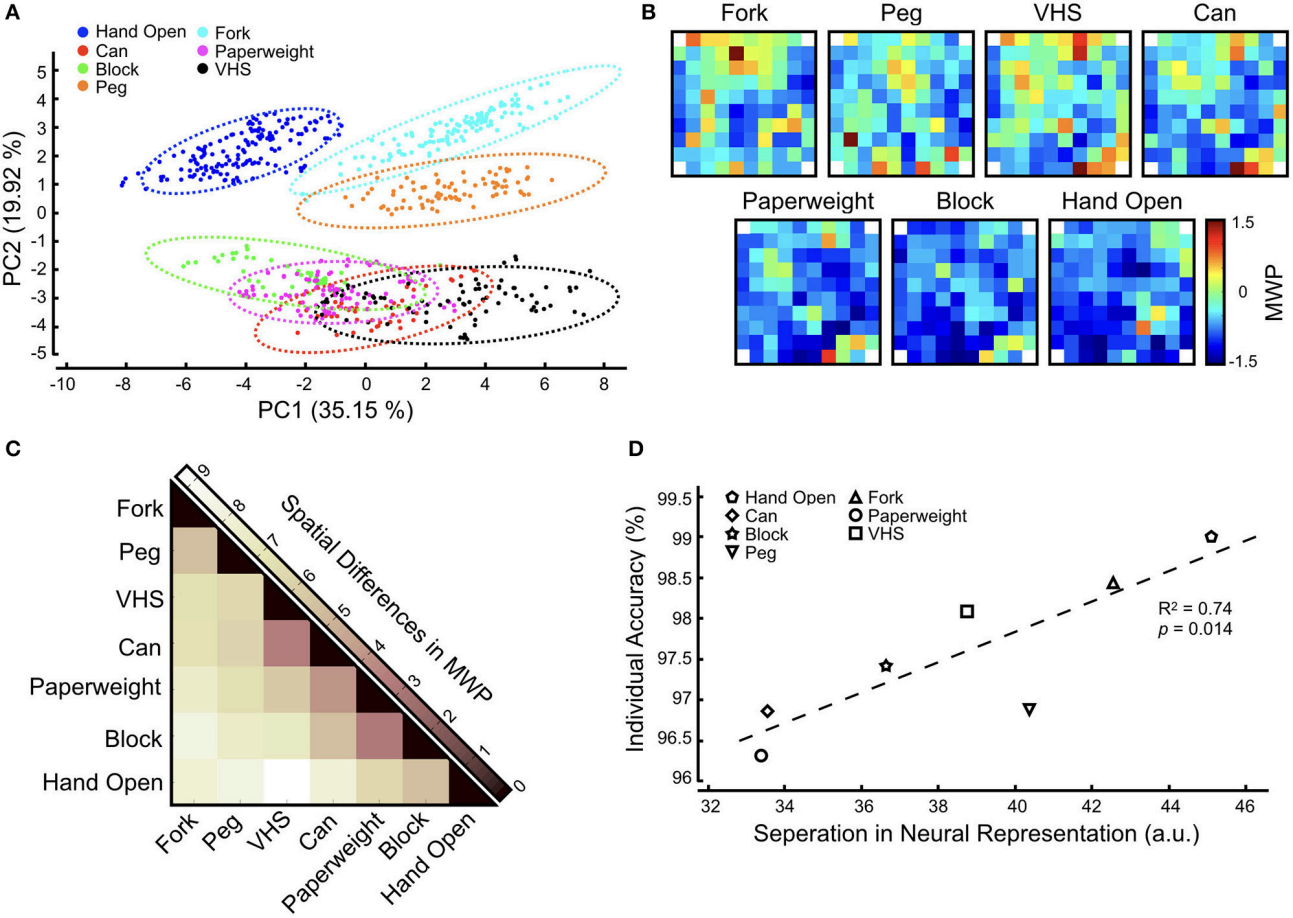
Neural representation of functional hand movements in the motor cortex. The participant was asked to attempt the cued hand movement. No FES was provided during this task so that neural data can be captured without any stimulation artifact. **(A)** Principal component analysis (PCA) of MWP activity shows clustering of neural activity for each hand movement during decoder activation for each functional movement. Dotted lines indicate a Gaussian mixture distribution model fit. **(B)** Heat maps of averaged MWP activity during neural decoder activation overlaid on the physical layout of the electrode array for each attempted hand movement. Corner reference (non-active) electrodes in the electrode array are labeled with white squares. **(C)** Heat map showing the pairwise Euclidean distances between vectorized MWP spatial patterns and highlights the separability in neural representation between different hand movements. Darker colors indicate that the neural representations are similar while lighter colors indicate that the representations are dissimilar. **(D)** Correlation between individual decoder accuracy and separation in neural representation (aggregate Euclidean distance for each movement) shows that higher neural discriminability leads to higher decoder accuracy. The trend line indicates a linear fit.

To further investigate these decoder misclassifications, we analyzed the neural modulation as the participant was asked to imagine the seven cued hand movements without FES (see section Methods). By not using the FES system, the neural modulation data we captured was free from stimulation and/or any movement induced artifacts. We applied a PCA to the MWP neural data to qualitatively illustrate clustering among different imagined hand movements (Figure 7A). We observed overlaps between the MWP clusters for the *Paperweight* and *Block* as well as the *VHS* and *Can*. Figure 7B shows the heat map of the average MWP for each imagined hand movement overlaid on the physical layout of the 96-channel cortical array showing the spatial distribution of MWP activity between different hand movements. To measure the discriminability of neural representations of different hand movements we computed the Euclidean distances between the MWP spatial distributions for all hand movements (Figure 7C). We found that the neural representation for the imagined *Paperweight* and *Block* grips as well as the *VHS* and *Can* grips were the most similar and might be one of the factors causing the decoder misclassifications observed during the GRT functional task. When compared to the results of the neural decoder training for the GRT task, we also observed a strong correlation (*R*^2^ = 0.74, *p* < *0.05*) between the individual decoder accuracy scores and the discriminability of neural representation of hand movements (Figure 7D). *Hand Open* movement had the most distinct neural representation and the highest individual movement accuracy while the *Paperweight* grasp had the least separated neural representation and the corresponding lowest movement accuracy.

## Discussion

The ability to successfully manipulate multiple real-world objects encountered during activities of daily living remains a key challenge limiting the practical applicability of BCI-controlled FES devices for people living with tetraplegia. In our previous studies, we demonstrated proof-of-concept that implanted BCI-transcutaneous FES technology can achieve motor control of a paralyzed upper limb after SCI (Bouton et al., 2016). We focused on demonstrating that differential control of individual wrist, finger, and hand movements could be achieved, but did not attempt to quantify or characterize the behavioral or neural features of motor control. In this study, we advance prior knowledge by applying standardized tasks developed for neuroprosthetic studies (GRT object manipulation) to the evaluation of system performance. In this way, we not only allow for comparison between our BCI-FES technology and other neuroprosthetics but also develop a new understanding of the strengths and limitations of the BCI-FES system. We showed that the participant in our study could train to use the BCI-controlled FES system to perform functional tasks that required dynamic integration of FES-enabled paralyzed hand/arm muscles with non-paralyzed shoulder/elbow muscles. The system enabled the participant to select the desired hand movement, out of the seven possible trained movements as well as a rest state available to him, using motor intent. The BCI-FES also enabled the participant to manipulate objects of different sizes, shapes, and weights with skilled, forceful grasps. In addition, our study revealed insights into the neural representation of hand movements in the motor cortex. We showed that stable representations of different hand movements can form in a very small area of the motor cortex under the implanted MEA. Furthermore, we demonstrated that discriminability between these neural representations can affect decoder performance.

Because the test objects varied widely in size and shape, the FES system was calibrated for each object to evoke a unique hand shape/movement pattern that provided grip force and dexterity to enable palmar, lateral, and tip-to-tip type grasps. The FES system calibration for each grasp involved precise targeting of separate muscle groups in the forearm to evoke specific finger movements (see Figure 3 and Figure S1 showing the target muscle groups for each grasp). The use of MWP as neural features for decoding provided a high-fidelity spatiotemporal neural modulation signal that was strongly correlated with neuronal spiking activity, and could be used to discriminate between different hand movements in real-time without the need for thresholding or explicit spike sorting (Figure 4). During decoder training, the participant attempted to evoke the correct grasp for a particular object from seven movement states (plus rest) available to him. The results from decoder training show that the participant was able to use the decoder to control the system with high accuracy—the individual accuracy scores for each movement were all >96% (Table 1) during different neural decoder training sessions across 4 days. Several groups have observed that neuronal states associated with different imagined hand movements may be represented discriminably in the human brain (Klaes et al., 2015; Bleichner et al., 2016; Leo et al., 2016). Our finding that multiple hand movements can be decoded reliably from the motor cortex is further validation of these observations. The consistently high accuracy of the decoders in classifying individual hand grasps not only indicates the robustness of the neural representations in the motor cortex, but also suggests that this modulation can be reliably leveraged for precision control of a FES neuro-orthotic device that can restore multiple hand-grasp functions. The results also highlight that for our trained participant the decoder training time for the multiple object manipulation task was limited to 12–15 min. These results have implications as high accuracy and minimal training time are features that are desirable for potential users of neuroprosthetic devices (Collinger et al., 2013).

We used the standardized GRT to demonstrate the participant's ability to successfully use the trained decoder to manipulate multiple objects. The use of a standardized measure of functional outcomes not only helped us better assess the performance of our system but also provided standardized reportable scores that can facilitate objective comparison with other similar technologies, help identify areas of improvements, enhance reproducibility of research, and aid in decision making for clinicians and potential end-users. The results show that using the BCI-FES system, the participant was able to evoke the correct movement to manipulate each of the six test objects using naturalistic grips (Figure 5 and Movie S2). It should be noted that the participant is able to transfer some of the objects on his own without using the BCI-FES system (see Movie S2, right panel showing the participant manipulating the objects on his own without FES). For example, the participant used adaptive strategies (such as biceps-mediated forearm supination with shoulder abduction/adduction) to easily grasp and release the *Peg* and *Block*. However, heavier objects that required a more forceful grip were difficult to transfer without the system (Figure 5B). Using the BCI-FES system, the participant was able to transfer the heavier objects (*VHS, Paperweight, Can, Fork*) and also showed significant improvement in transfer times (Figure 5C).

The transfer speed using the BCI-FES system during the GRT compares favorably to those reported for other BCI-FES systems. For example, our participant transferred the *Paperweight* at a rate of 4.7 ± 1.2 s per transfer compared to ~36 s it took a participant to transfer the *Paperweight* using the EEG-Freehand system (Müller-Putz et al., 2005). It should be noted that the neurologic level of the participant in Muller-Putz et al. (C5 ASI A with residual shoulder and elbow movements) is functionally similar to the neurologic level of the participant in our study (C5 ASI A with residual shoulder and elbow movements). While the participant in our study had a twitch of wrist extension (yielding a zone of partial preservation to C6), he was not able to elicit the tenodesis grip of a person with a C6 neurologic level. The improved performance on the GRT with our BCI-FES system carries further significance as compared to the EEG-Freehand system where the participant only has a single grasp available, our participant had seven hand functions available to him and he can voluntarily choose the one which provides him the optimal grip for the target object. The transfer speed with our system also compares favorably with a BCI-controlled robotic arm used by paralyzed individuals—for example, a transfer rate of 6–10 transfers per minute for the *Block* vs. 0.1–1 transfers per minute reported for the BCI-robotic arm used to transfer similar sized blocks during the Box and Block (BBT) test (Wodlinger et al., 2015).

Analysis of the participant's neural modulation, as he imagined different hand movements, revealed interesting insights into the neural representations of hand grasps in the motor cortex. The PCA revealed overlaps in the MWP clusters of the grips for *Paperweight*–*Block* and the *Can*–*VHS* (Figure 7A). The similarities in neural representation for hand grasps were consistent with the spatial distribution of MWP on the cortical array where we observed a group of channels that appear to modulate similarly between these grasps (Figure 7B). The analysis of the differences in MWP modulation confirmed that the neural representation of the imagined *Block* and *Paperweight* as well as the *Can* and *VHS* grasps were, indeed, the most similar of all grasps (Figure 7C). This was consistent with hand morphology observed during performance of the grips, with *Block* and *Paperweight* grasp patterns being a synergy of lateral key and tip-to-tip precision grips and *Can* and *VHS* grasp patterns representing versions of a palmar power grip. This similarity in the neural representations for certain hand grasps may be one of the factors underlying the misclassification in decoding that we observed during the GRT test blocks. Other groups have made similar observations. For example, Leo et al. observed a clustering of neuronal representations based on postural differences in hand shapes (i.e., precision grasps and power grasps) which in turn affected the ability to correctly classify these hand shapes during decoding (Leo et al., 2016). Similarly, Bleichner et al. used an ECoG-based BCI to classify four different hand gestures and noted that the gestures that correlated strongest in neural representation were misclassified most often (Bleichner et al., 2016). The results not only expand on the total number of hand movements for which a stable representation could be observed in the motor cortex, but also show that it is possible to study and decode neural representations in a very small area under the implanted MEA. Regardless, our findings that there are overlaps between the MWP spatial patterns for some hand movements highlights that additional neural features (such as signal propagation or phase) and/or other decoding algorithms (such as deep learning algorithms) might need to be explored to expand the repertoire of hand functions that can be reliably decoded using a single MEA.

Interestingly, the neural representation for *Hand Open* in our experiments was the most distinct from the six other hand grasps (Figure 7C). We hypothesize that this is due to the *Hand Open* posture being morphologically distinct from all other grasps. In addition, the FES pattern for *Hand Open* was primarily over wrist and hand extensors, while the FES pattern for grasps often included both flexor and extensor muscle compartments. Therefore, somatosensory and muscle stretch receptor feedback from stimulation of sensate areas could have propagated to the motor cortex and differentially influenced neural activation patterns in *Hand Open* vs. grasp states. Not surprisingly therefore, during neural decoder training we observed the highest accuracy when the participant attempted a *Hand Open* movement (Table 1). Overall, there was a strong correlation between the discriminability in neural representation of different hand states and the corresponding decoding accuracy (Figure 7D). It should be noted that, compared to GRT test blocks, we rarely observed this misclassification among hand movements during the decoder training. This may be due to the differences in how the training and GRT test blocks are performed. First, in contrast to training blocks, the participant does not receive visual cues to initiate, sustain, and terminate the grasp during the GRT test blocks. Second, the decoder training is more structured and motor imagery is more consistent and deliberate as the participant must grasp and transfer the cued object once during the cue period. The GRT test block, however, may be more challenging for the participant because he must quickly and repeatedly activate and deactivate decoders to transfer objects as many times as he can in a 30-s test window. We believe that it is the combination of the lack of reinforcing visual cues, and the rapid task switching during the “beat the clock” condition of the GRT that increases the misclassification probability of the decoder for grasps with most similar neural representations.

Enabling grasp and manipulation abilities using BCI-FES technology is challenging compared to, for example, a 3-D reaching task or individual finger/joint movement as it not only requires high fidelity control signals and strategies (Schaffelhofer et al., 2015), but may also require additional sensorimotor information related to the shape of the target object that may be needed to preshape the hand correctly (Leo et al., 2016). In addition, a reaching task in space involves coordinating only three degrees of freedom (DOF) whereas control of an anthropomorphic hand requires control of 23 DOF (Vargas-Irwin et al., 2010) thereby increasing the complexity of the problem. Not surprisingly therefore, there are only a few reports of successful demonstration of BCI-enabled hand grasp, most of which were limited by the number of functional hand movements that could be enabled (Bouton et al., 2016; Sharma et al., 2016; Ajiboye et al., 2017; Friedenberg et al., 2017). Not only are the number of hand functions regained by our tetraplegic participant to manipulate objects substantially more than achieved by any previous study of FES devices, but we also show that this improvement did not come at the cost of accuracy, speed, or training time. Our results also have implications beyond reanimation in tetraplegia. The enhanced understanding of the neural representation of hand gestures in the human brain and the ability to accurately decode these movements can provide a novel control signal for the development of other BCI tools; for instance, communication based on sign language (Bleichner et al., 2016). Another advantage of using an intuitive BCI paired with real-time FES is the potential to promote synaptic neuroplasticity in the cortico-spinal tract (McGie et al., 2015) or to promote neuroprosthetic “learning” in the motor cortex (Ganguly et al., 2011).

It is important to note that this study is limited to one participant who had over two years of experience using the BCI to evoke hand and forearm states prior to performing GRT testing. Novice BCI users may take longer to achieve the same level of hand dexterity for object manipulation as described here. In addition, the transcutaneous FES cuff used in this study is designed to stimulate the paralyzed forearm muscles to control hand and wrist movements. It is therefore best suited to persons with C5 or lower levels of tetraparesis and who have some residual shoulder and biceps movements. Testing to assess whether the FES cuff can be used along with shoulder and triceps stimulation or a gravity assisting device in SCI patients with higher level of injury remains to be investigated. Our device was also limited by the lack of thenar (base of thumb) muscle stimulation, limiting the quality of hand grasps for small objects requiring precision grips (no objects of this type are represented in the GRT). The need for daily retraining of the decoders is another limitation of the current system that will need to be overcome in order to reduce setup time and facilitate translation of the device for daily use.

In summary, our BCI-FES neuro-orthotic device significantly improves upon the state-of-the-art for assistive devices capable of meeting tetraplegic individual's desired priorities of restoring multiple, voluntary, and naturalistic hand functions. We also demonstrate that our BCI-FES system can enable functional, skilled hand grasps that can generate adequate force to manipulate everyday objects with high-precision and practical speed. The fact that the participant could use the system to perform functional tasks ~900 days post-implantation further highlights the translational potential of our system. Future directions include addressing system limitations to make the next generation BCI-FES robust to daily neural signal variability, portable, wearable, with more electrodes and sensors, and less obtrusive to further facilitate clinical translation.

## Author contributions

GS conceptualized the study; GS, HB, SC, and MB designed the scientific experiments; SC, MZ, GS, and MB performed research; DF and MS developed the decoding algorithms; NA contributed to developing the stimulation hardware and software; AR performed the surgery; SC, MZ, NS, and GS analyzed data; WM and MB were involved in participant recruitment; GS and SC wrote the manuscript. All authors contributed to editing the manuscript.

### Conflict of interest statement

The authors declare that the research was conducted in the absence of any commercial or financial relationships that could be construed as a potential conflict of interest.

## Supplementary material

Figure S1 FES parameters and target muscle groups for each hand movement. **(A)** Representative image showing spatial mapping of active electrodes on the lower and upper FES cuffs used to enable the *Can* grasp. Red and black electrodes indicate high-side and low-side electrodes, respectively. **(B)** Active electrodes, stimulation amplitude, and targeted muscle groups for each hand movement. L, Lower cuff; U, Upper cuff.

Figure S2 Mean number of failed attempts on the GRT with and without the BCI-FES system. The participant had fewer failures with the use of the BCI-FES system. ^*^The *Can* transfer required activation of two hand movements—*Hand Open* and *Can* grasp.

Movie S1 Representative video of the participant manipulating the GRT objects during decoder training. Test objects were placed in front of the participant and he was cued to start and stop by the small virtual hand on the monitor in front of him.

Movie S2 Representative video of the participant performing the GRT for all objects with and without the BCI-FES system. The participant was verbally cued to start and stop the 30 s GRT trial.

## References

[B1] Ajiboye A. B. Willett F. R. Young D. R. Memberg W. D. Murphy B. A. Miller J. P.. (2017). Restoration of reaching and grasping movements through brain-controlled muscle stimulation in a person with tetraplegia: a proof-of-concept demonstration. Lancet 389, 1821–1830. 10.1016/S0140-6736(17)30601-3 28363483 PMC5516547

[B2] Anderson K. D. (2004). Targeting recovery: priorities of the spinal cord-injured population. J. Neurotrauma 21, 1371–1383. 10.1089/neu.2004.21.1371 15672628

[B3] Baranauskas G. (2014). What limits the performance of current invasive brain machine interfaces? Front. Syst. Neurosci. 8:68. 10.3389/fnsys.2014.00068 24808833 PMC4010778

[B4] Blabe C. H. Gilja V. Chestek C. A. Shenoy K. V. Anderson K. D. Henderson J. M. (2015). Assessment of brain-machine interfaces from the perspective of people with paralysis. J. Neural Eng. 12:043002. 10.1088/1741-2560/12/4/043002 26169880 PMC4761228

[B5] Bleichner M. G. Freudenburg Z. V. Jansma J. M. Aarnoutse E. J. Vansteensel M. J. Ramsey N. F. (2016). Give me a sign: decoding four complex hand gestures based on high-density ECoG. Brain Struct. Funct. 221, 203–216. 10.1007/s00429-014-0902-x 25273279 PMC4720726

[B6] Bouton C. E. Shaikhouni A. Annetta N. V. Bockbrader M. A. Friedenberg D. A. Nielson D. M.. (2016). Restoring cortical control of functional movement in a human with quadriplegia. Nature 533, 247–250. 10.1038/nature17435 27074513

[B7] Chadwick E. K. Blana D. Simeral J. D. Lambrecht J. Kim S. P. Cornwell A. S.. (2011). Continuous neuronal ensemble control of simulated arm reaching by a human with tetraplegia. J. Neural Eng. 8:034003. 10.1088/1741-2560/8/3/034003 21543840 PMC3608269

[B8] Collinger J. L. Boninger M. L. Bruns T. M. Curley K. Wang W. Weber D. J. (2013). Functional priorities, assistive technology, and brain-computer interfaces after spinal cord injury. J. Rehabil. Res. Dev. 50, 145–160. 10.1682/JRRD.2011.11.0213 23760996 PMC3684986

[B9] Ethier C. Miller L. E. (2015). Brain-controlled muscle stimulation for the restoration of motor function. Neurobiol. Dis. 83, 180–190. 10.1016/j.nbd.2014.10.014 25447224 PMC4412757

[B10] Friedenberg D. Schwemmer M. Landgraf A. Annetta N. Bockbrader M. Bouton C.. (2017). Neuroprosthetic-enabled control of graded arm muscle contraction in a paralyzed human. Sci. Rep. 7:8386. 10.1038/s41598-017-08120-9 28827605 PMC5567199

[B11] Ganguly K. Dimitrov D. F. Wallis J. D. Carmena J. M. (2011). Reversible large-scale modification of cortical networks during neuroprosthetic control. Nat. Neurosci. 14, 662–667. 10.1038/nn.2797 21499255 PMC3389499

[B12] Humber C. Ito K. Bouton C. (2010). Nonsmooth formulation of the support vector machine for a neural decoding problem. arXiv:1012.0958.

[B13] ICCP (2017). International Campaign for Cures of Spinal Cord Injury Paralysis. 10.1038/sj.sc.3101597 14968108

[B14] Klaes C. Kellis S. Aflalo T. Lee B. Pejsa K. Shanfield K.. (2015). Hand shape representations in the human posterior parietal cortex. J. Neurosci. 35, 15466–15476. 10.1523/JNEUROSCI.2747-15.2015 26586832 PMC4649012

[B15] Lauer R. T. Peckham P. H. Kilgore K. L. (1999). EEG-based control of a hand grasp neuroprosthesis. Neuroreport 10, 1767–1771. 10.1097/00001756-199906030-00026 10501572

[B16] Leo A. Handjaras G. Bianchi M. Marino H. Gabiccini M. Guidi A.. (2016). A synergy-based hand control is encoded in human motor cortical areas. Elife 5:e13420. 10.7554/eLife.13420 26880543 PMC4786436

[B17] Mallat S. (1998). A Wavelet Tour of Signal Processing. Burlington, MA: Academic Press.

[B18] Márquez-Chin C. Popovic M. R. Cameron T. Lozano A. M. Chen R. (2009). Control of a neuroprosthesis for grasping using off-line classification of electrocorticographic signals: case study. Spinal Cord 47, 802–808. 10.1038/sc.2009.41 19381156

[B19] McGie S. C. Zariffa J. Popovic M. R. Nagai M. K. (2015). Short-term neuroplastic effects of brain-controlled and muscle-controlled electrical stimulation. Neuromodulation 18, 233–240. 10.1111/ner.12185 24802088

[B20] Müller-Putz G. R. Scherer R. Pfurtscheller G. Rupp R. (2005). EEG-based neuroprosthesis control: a step towards clinical practice. Neurosci. Lett. 382, 169–174. 10.1016/j.neulet.2005.03.021 15911143

[B21] Nas K. Yazmalar L. Sah V. Aydin A. Ones K. (2015). Rehabilitation of spinal cord injuries. World J. Orthop. 6, 8–16. 10.5312/wjo.v6.i1.8 25621206 PMC4303793

[B22] Pfurtscheller G. Muller G. R. Pfurtscheller J. Gerner H. J. Rupp R. (2003). “Thought”–control of functional electrical stimulation to restore hand grasp in a patient with tetraplegia. Neurosci. Lett. 351, 33–36. 10.1016/S0304-3940(03)00947-9 14550907

[B23] Schaffelhofer S. Agudelo-Toro A. Scherberger H. (2015). Decoding a wide range of hand configurations from macaque motor, premotor, and parietal cortices. J. Neurosci. 35, 1068–1081. 10.1523/JNEUROSCI.3594-14.2015 25609623 PMC6605542

[B24] Sharma G. Friedenberg D. A. Annetta N. Glenn B. Bockbrader M. Majstorovic C.. (2016). Using an artificial neural bypass to restore cortical control of rhythmic movements in a human with quadriplegia. Sci. Rep. 6:33807. 10.1038/srep33807 27658585 PMC5034342

[B25] Simpson L. A. Eng J. J. Hsieh J. T. Wolfe D. L. (2012). The health and life priorities of individuals with spinal cord injury: a systematic review. J. Neurotrauma 29, 1548–1555. 10.1089/neu.2011.2226 22320160 PMC3501530

[B26] Snoek G. J. IJzerman M. J. Hermens H. J. Maxwell D. Biering-Sorensen F. (2004). Survey of the needs of patients with spinal cord injury: impact and priority for improvement in hand function in tetraplegics. Spinal Cord 42, 526–532. 10.1038/sj.sc.3101638 15224087

[B27] Stroh-Wuolle K. Van Doren C. L. Thrope G. B. Keith M. W. Peckham P. H. (1994). Development of a quantitative hand grasp and release test for patients with tetraplegia using a hand neuroprosthesis. J. Hand Surg. 19, 209–218. 10.1016/0363-5023(94)90008-6 8201183

[B28] Vargas-Irwin C. E. Shakhnarovich G. Yadollahpour P. Mislow J. M. Black M. J. Donoghue J. P. (2010). Decoding complete reach and grasp actions from local primary motor cortex populations. J. Neurosci. 30, 9659–9669. 10.1523/JNEUROSCI.5443-09.2010 20660249 PMC2921895

[B29] Wodlinger B. Downey J. E. Tyler-Kabara E. C. Schwartz A. B. Boninger M. L. Collinger J. L. (2015). Ten-dimensional anthropomorphic arm control in a human brain-machine interface: difficulties, solutions, and limitations. J. Neural Eng. 12:016011. 10.1088/1741-2560/12/1/016011 25514320

