# Supplementary material for: Dexterous Control of Seven Functional Hand Movements Using Cortically-Controlled Transcutaneous Muscle Stimulation in a Person With Tetraplegia
Source: Front Neurosci. 2018 Apr 4;12:208. doi: 10.3389/fnins.2018.00208 (PMC5893794; doi:10.3389/fnins.2018.00208)
Supplement: Figure S1 — FES parameters and target muscle groups for each hand movement. (A) Representative image showing spatial mapping of active electrodes on the lower and upper FES cuffs used to enable the Can grasp. Red and black electrodes indicate high-side and low-side electrodes, respectively. (B) Active electrodes, stimulation amplitude, and targeted muscle groups for each hand movement. L, Lower cuff; U, Upper cuff. [file Image1.PDF]

A

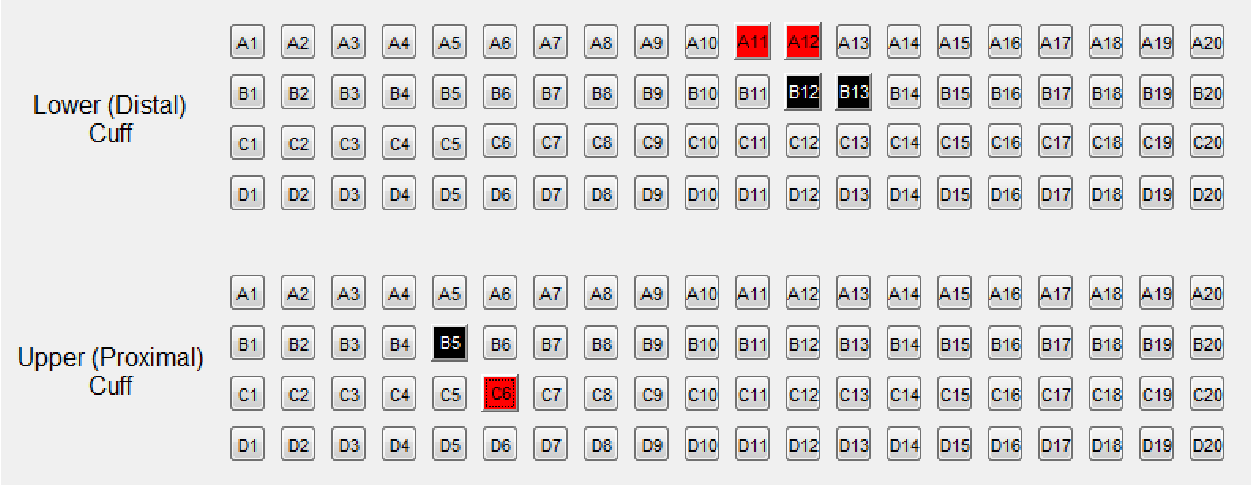

B

Hand Movement/ Grasp	Active Electrodes	Stimulation Amplitude (mA)	Targeted Forearm Muscle Groups
Hand Open	LB8, LC7, LC14, UA8, UC7, UC8, UD8, UD9	12.26	Extensor digitorum communis; Extensor pollicis longus
Can	LA11, LA12, LB12, LB13, UB5, UC6	13.00	Flexor pollicis longus; Flexor digitorum profundus and sublimis; Extensor carpi radialis longus
VHS	LA12, LA13, LB13, LB14, UA3, UB4, UV11, UC12, UD11	11.56	Abductor pollicis longus; Flexor digitorum profundus: Extensor carpi radialis longus
Block	LB12, LB13, LC11, LC12, UA15, UB10, UB16, UC9	8.80	Flexor pollicis longus; Flexor digitorum profundus and sublimis
Peg	LB1, LB14, LC13, LC14, UA15, UB10, UB16, UC7, UC9, UD6	11.00	Abductor pollicis longus; Flexor digitorum profundus and sublimis
Fork	LC4, LC5, LC14, LD13, UA15, UB4, UB5, UB14	12.36	Flexor pollicis longus; Flexor digitorum profundus and sublimis; Extensor carpi radialis
Paperweight	LA1, LA12, LB1, LB13, UA15, UB5, UB16, UC5	15.00	Abductor pollicis longus; Flexor digitorum profundus and sublimis; Extensor carpi radialis longus

Supplementary Figure S1. FES parameters and target muscle groups for each hand movement. (**A**) Representative image showing spatial mapping of active electrodes on the lower and upper FES cuffs used to enable the *Can* grasp. Red and black electrodes indicate high-side and low-side electrodes respectively. (**B**) Active electrodes, stimulation amplitude, and targeted muscle groups for each hand movement. L – Lower cuff, U – Upper cuff.
