# Supplementary material for: Dexterous Control of Seven Functional Hand Movements Using Cortically-Controlled Transcutaneous Muscle Stimulation in a Person With Tetraplegia
Source: Front Neurosci. 2018 Apr 4;12:208. doi: 10.3389/fnins.2018.00208 (PMC5893794; doi:10.3389/fnins.2018.00208)
Supplement: Figure S2 — Mean number of failed attempts on the GRT with and without the BCI-FES system. The participant had fewer failures with the use of the BCI-FES system. *The Can transfer required activation of two hand movements—Hand Open and Can grasp. [file Image2.PDF]

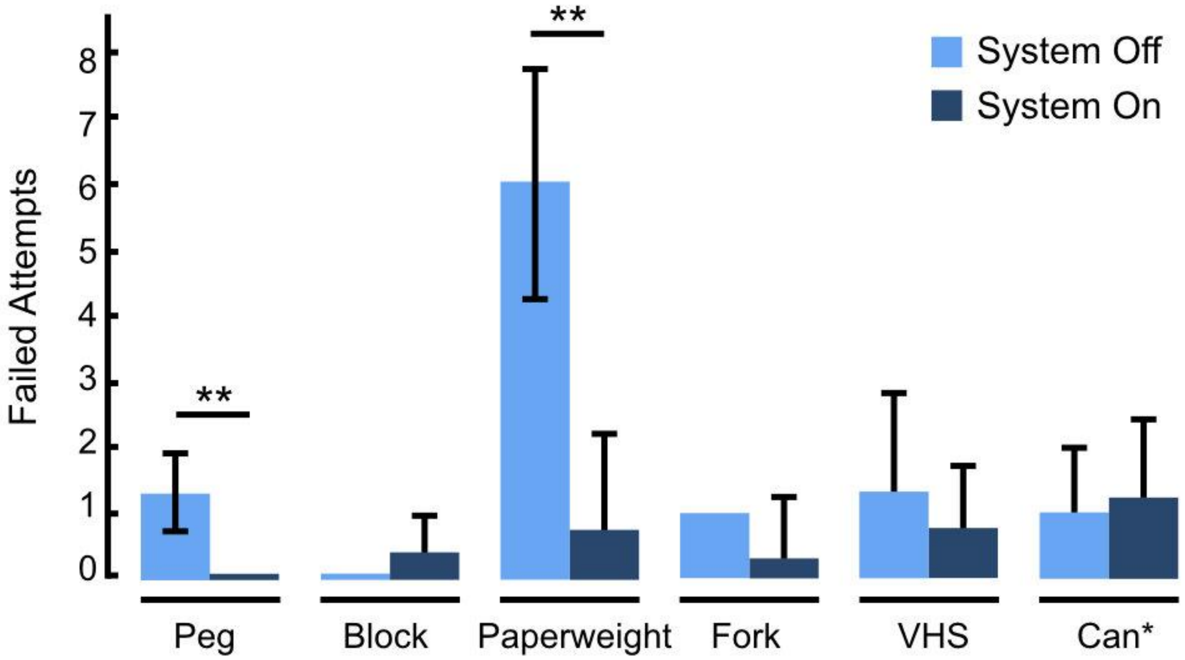

Fig. S2. Mean number of failed attempts on the GRT with and without the BCI-FES system. The participant had fewer failures with the use of the BCI-FES system. *The *Can* transfer required activation of two hand movements – *Hand Open* and *Can* grasp.
